# Reviving lower body negative pressure as a countermeasure to prevent pathological vascular and ocular changes in microgravity

**DOI:** 10.1038/s41526-020-00127-3

**Published:** 2020-12-17

**Authors:** Katie M. Harris, Lonnie G. Petersen, Tobias Weber

**Affiliations:** 1 Faculty of Medicine, Memorial University of Newfoundland, St. John’s, NL Canada; 2 Department of Mechanical and Aerospace Engineering, University of California San Diego, San Diego, CA USA; 3 Department of Radiology, University of California San Diego, San Diego, CA USA; 4 Department of Biomedical Science, University of Copenhagen, Copenhagen, Denmark; 5 Space Medicine Team (HRE-OM), European Astronaut Centre (EAC), European Space Agency (ESA), Köln, Germany; 6 KBR GmbH, Köln, Germany

**Keywords:** Eye manifestations, Risk factors, Eye diseases, Physiology, Cardiovascular diseases

## Abstract

Mitigation of spaceflight-related pathologies such as spaceflight-associated neuro-ocular syndrome (SANS) and the recently discovered risk of venous thrombosis must happen before deep space exploration can occur. Lower body negative pressure (LBNP) can simulate gravitational stress during spaceflight that is likely to counteract SANS and venous thrombosis, but the ideal dose and method of delivery have yet to be determined. We undertook a review of current LBNP literature and conducted a gap analysis to determine the steps needed to adapt LBNP for in-flight use. We found that to use LBNP in flight, it must be adapted to long time duration/low pressure use that should be compatible with crew activities. A lack of understanding of the etiology of the pathologies that LBNP can counteract hinders the application of LBNP as a countermeasure during spaceflight. Future research should aim at filling the knowledge gaps outlined in this review.

## Introduction

Future human missions beyond low Earth orbit (LEO) require space agencies to revise their operational procedures due to increased challenges in effective ground-space communication, down-sizing of space vehicles, and inability to return quickly to Earth during medical emergencies. As a consequence, human deep space exploration missions will require more crew autonomy as well as robust, reliable, and safe procedures to mitigate unwanted effects of prolonged microgravity (µG) exposure.

During exposure to µG, fluid from the legs moves into the thorax and head, causing redistribution of fluid in the tissues and vessels of the body^1^. The body adapts to this fluid shift by increasing urination which decreases plasma volume; however, increased cardiac output and stroke volume, and decreased mean arterial pressure are still observed, among other effects, indicating the limitations of the body’s ability to adapt to µG^2,3^. Cephalad fluid shift is an inevitable consequence of weightlessness and likely associated with many negative health outcomes, some of which have been characterized through ground-based analogs such as posture change, bed rest, water (and dry) immersion, and parabolic flight^4–6^. In particular, effects on cardiac system and orthostatic tolerance have been well explored^1,3,7–9^, but existing countermeasures to mitigate these changes have been found to be largely inadequate, either in terms of time, safety, efficacy, or practicality^10–13^. Orthostatic tolerance decreases after just days in-flight^14^, yet astronauts will have to function autonomously after landing on the Moon or Mars without a safety medical crew for assistance. Therefore, adequate orthostatic tolerance maintenance in-flight is a key factor for successful and safe planetary surface exploration.

Other pathologies are still being discovered and characterized during long duration LEO missions. Spaceflight-associated neuro-ocular syndrome (SANS) requires mitigation before planetary missions as SANS poses a significant risk both to the individual crew and the overall mission success^15^. Venous thrombosis was recently identified during a long duration mission^16,17^, and highlighted the risks regarding treating emergent medical conditions in-flight. Pathologies related to altered hemodynamics, therefore, also require adequate mitigation strategies before planetary missions in deep space (humanresearchroadmap.nasa.gov).

Most importantly, countermeasures that are able to mitigate these effects of spaceflight need to be developed before long duration spaceflight can occur. Lower body negative pressure (LBNP) is a promising candidate. By simulating gravitational stress during prolonged periods of µG exposure, the body systems that have evolved under 1 G can be maintained and adaptations to µG that are maladaptive upon return to Earth, such as cerebral and ocular restructuring, can be avoided. Pathologies that are specifically linked to fluid shifting in µG are particularly likely to be mitigated by regular exposure to simulated gravitational stress through LBNP. In order of strongest evidence, the possible pathologies that may be mitigated by LBNP are:

1. Orthostatic intolerance prior to re-entry^8,18–20^.
2. SANS, given the possibility of limiting cephalad fluid shifting consequently limiting cerebral and ocular restructuring^21–23^.
3. Venous thrombosis, by potentially restoring normal venous hemodynamics^16,24,25^.
4. Cardiovascular degradation, as LBNP can possibly maintain heart function as well as blood vessel function and reflexes^26,27^.
5. Musculoskeletal degradation, as LBNP generates mechanical load that can be used in combination with exercise^26–30^.

The aim of this review is therefore to highlight potential benefits of LBNP related to risks associated with long duration spaceflight, to reveal knowledge gaps, and provide research recommendations to fill these knowledge gaps. Considering their operational relevance, the focus of this review will be on SANS, deep vein thrombosis (DVT) and venous pooling, both as its own risk and as a possible causative factor for SANS.

## Review methodology

A comprehensive narrative review was undertaken to survey the papers relevant to the use of LBNP as a long duration countermeasure for SANS and venous flow pathologies. This methodology was chosen specifically to review the recent changes in the study of SANS and venous flow pathologies within the theoretical context of the applicability of LBNP. Although a full systematic review was outside the scope of this project, survey tools from the Cochrane handbook for systematic reviews (https://training.cochrane.org/handbook) were used to increase the scientific rigor and to standardize the assessment of the included studies.

The search strategy involved keyword searches in PubMed and Google Scholar. Key words included “Lower Body Negative Pressure”, “Countermeasures”, “Venous Flow”, “Thrombosis”, “Long Duration Spaceflight”, “SANS”, “VIIP”, “Venous Hemodynamics”, and “Cerebral remodeling” combined in Boolean search format. Relevant space actors’ repositories were also searched, such as NASA’s Technical Reports Server (https://ntrs.nasa.gov/search.jsp?R=20050189209), and ESA’s experiment archive (http://eea.spaceflight.esa.int/portal/). Manuscripts were limited to fully accessible peer reviewed papers that were published in or translated into English, which involved studies using human participants or relevant reviews of technology or pathologies.

**Fig. 1:**
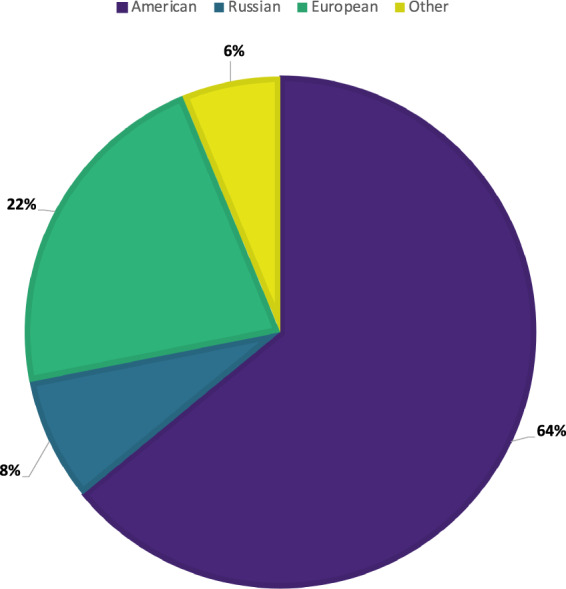
National distribution of sources. Country of origin of the papers included in this review.

**Fig. 2:**
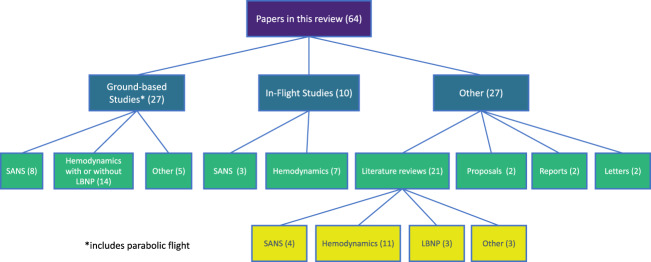
Types of sources included in this review. Distribution of included papers by type of study, type of article, and topic.

Distribution of the source country of the included papers is shown in Fig. 1, and the type of studies included in this review are shown in Fig. 2. Further breakdown of included studies is shown in Table 1 below, using the PICOS system.

**Table 1:** Population, intervention, control, outcome, and study design of included in-flight and ground-based studies.

Paper	Population	Intervention	Control conditions	Outcome	Study design
Anderson et al.^62^	Healthy volunteers (17)	Short arm centrifuge	Standing and supine	Reduction in intraocular pressure	Crossover
Arbeille et al.^1^	Healthy volunteers (12) in two ground-based studies, and astronauts (1 and 2, respectively) in two in-flight studies	28-d HDT and LBNP, and 30-d HDT with LBNP + exercise for the ground-based studies. Thigh cuffs for one astronaut in the second in-flight study	No LBNP and no LBNP + exercise for the ground-based studies, no thigh cuffs for the second in-flight study	Orthostatic tolerance (ground based) Vascular tone (in-flight)	Randomized Control Trial for the ground-based studies, Case Study and Case-Control Study for the in-flight studies
Arbeille et al.^25^	Healthy female volunteers (24)	60-d HDT with LBNP + exercise or nutrition supplement	No LBNP + exercise or nutrition supplement	Cardiovascular response to tilt testing	Randomized Control Trial
Arbeille et al.^33^	Astronauts (10)	Spaceflight	Ground	Jugular vein, portal vein, femoral vein, tibial vein, and gastrocnemius vein cross-sectional area	Cohort Study
Arbeille et al.^5^	Healthy volunteers (10)	2 h of dry immersion	Rest	Jugular vein, portal vein and thyroid volume. middle cerebral vein velocity, intracranial pressure	Crossover Study
Auñón-Chancellor et al.^17^	Astronaut (1)	Spaceflight	Ground	Venous flow	Case Study
Baisch et al.^8^	Astronauts (6)	Spaceflight	Ground	Heart rate, blood pressure, and total peripheral resistance	Cohort Study
Baranov et al.^60^	Healthy male volunteers (6)	7-d HDT with LBNP and negative pressure respiration	HDT without LBNP, and HDT with LBNP	Orthostatic tolerance	Randomized Control Trial
Breit et al.^53^	Healthy volunteers (9)	LBNP	HUT to 90°	Cutaneous blood flow	Crossover Study
Chen et al.^63^	31 patients with IIH	Disease state	Healthy state	Visual acuity loss in IIH	Retrospective Observational Study
Custaud et al.^10^	Healthy male volunteers (8)	7-d HDT with thigh cuffs	HDT without thigh cuffs	Orthostatic tolerance, plasma volume, autonomic influences, and baroreflex sensitivity	Crossover Study
Güell et al.^20^	Healthy volunteers (5)	30-d bed rest with LBNP	Bed rest without LBNP	Orthostatic tolerance	Crossover Study
Halliwill et al.^24^	Healthy volunteers (16)	Graded LBNP with anti-shock trousers	No anti-shock trousers	Heart rate and mean arterial pressure	Randomized Control Trial
Hirsch et al.^54^	Healthy volunteers (2)	Graded LBNP	Rest state	Blood flow and pulse pressure changes	Crossover Study
Iwasaki et al.^50^	Astronauts (6)	LBNP (Space), HUT (Earth) and spontaneous breathing	Before, during, and post flight	Cerebral autoregulation by means of mean arterial pressure and cerebral blood flow velocity	Cohort Study
Kotovskaia and Fomina^31^	Astronauts (26)	Spaceflight	Ground	Hemodynamic parameters, orthostatic tolerance, leg vein capacity	Retrospective Observational Study
Kuriyama et al.^64^	20 patients with iNPH	Valsalva maneuver	13 healthy controls	Retrograde jugular venous flow	Randomized Control Trial
Laurie et al.^58^	Healthy volunteers (11)	30-d HDT in mild hypercapnic environment	Normal conditions	Optic disc edema	Cohort Study
Lawley et al.^51^	Volunteers with Ommaya reservoirs (8)	Acute zero gravity (parabolic flight)	Upright and supine position	Intracranial pressure	Cohort Study
Lee et al.^26^	Healthy female volunteers (24)	60-d bed rest with exercise or protein supplementation	Bed rest without countermeasures	Strength, endurance, and leg lean mass	Randomized Control Trial
Levine et al.^7^	Healthy male volunteers (13)	LBNP	Rest state	Systemic flow, regional forearm flow, blood pressure, calculated system and vascular resistances	Cohort Study
Mader et al.^40^	Astronauts (7 heavily documented, 300 additional)	Spaceflight	Ground	Ophthalmic findings (optic disc edema, globe flattening, choroidal folds, cotton wool spots, nerve fiber layer thickening, decreased near vision)	Retrospective Observational Study
Marshall-Goebel et al.^16^	Astronauts (11)	Spaceflight and LBNP	Ground (seated, supine, and 15 HDT)	Internal jugular vein measurements (pressure and flow)	Cohort Study
Marshall-Goebel et al.^22^	Healthy male volunteers (9)	Five HDT conditions (6, 12, 18) and 12 with LBNP, 12 with 1% with CO_2_	Rest state	Volumetric scan of the cranium, and intracranial CSF volume and ONSD	Crossover Study
Norsk et al.^32^	Astronauts (8)	Spaceflight	Ground	Ambulatory brachial arterial pressures	Cohort Study
Ogoh et al.^49^	Healthy volunteers (9)	Hyperventilation and 6% CO_2_ inhalation while seated	Supine	Arterial and venous flow responses in anterior and posterior parts of the brain	Crossover Study
Petersen et al.^4^	Healthy volunteers (8)	Microgravity in 20 s bursts (parabolic flight) with thigh cuffs	Microgravity without thigh cuffs	Cardiac output	Crossover Study
Petersen et al.^52^	Neurosurgical patients undergoing ICP monitoring (9)	Tilt testing	Supine	Intracranial pressure, cerebral perfusion pressure, and mean arterial pressure	Unclear*
Petersen et al.^28^	Healthy volunteers (8)	Mobile LBNP suit	Rest	Cardiovascular response (Nexfin), internal jugular venous cross-sectional area (IJVa), and ground reaction forces	Crossover Study
Petersen et al.^21^	Healthy volunteers (10)	LBNP while supine and 15 HDT	Supine	Intracranial pressure and blood pressure	Crossover Study
Qvarlander et al.^41^	Patients with NPH (27)	HUT	Supine	Intracranial pressure	Crossover Study
Roberts et al.^42^	Astronauts (18 LDSF, 16 SDSF)	Spaceflight	Ground	Extent of narrowing of CSF spaces and displacement of brain structure	Crossover Study
Smith et al.^27^	Healthy female volunteers (16)	60-d HDT + exercise	HDT without exercise	Hip and leg bone mineral density	Randomized Control Trial
Sun et al.^13^	Healthy male volunteers (12)	21-d HDT with LBNP	HDT without LBNP	Orthostatic tolerance	Randomized Control Trial
Watenpaugh et al.^61^	Healthy subjects (15)	Short arm and long arm centrifuge, tilt, and LBNP	Rest state	Regional skin blood flow	Crossover Study
Watenpaugh et al.^19^	Healthy identical twins, 14 females, 16 males	30-d HDT + exercise in LBNP	HDT without exercise in LBNP	Orthostatic tolerance	Case-Control Study
Watkins et al.^23^	Healthy volunteers (15)	HDT with and without LBNP, and sitting position	Supine	Non-invasive intracranial pressure and internal jugular vein cross-sectional area	Crossover Study

*HDT* head down tilt, *CSF* cerebrospinal fluid, *ONSD* optic nerve sheath diameter.

*Indicates that the study design is not explicitly described in the text.

## Pathologies that may be mitigated by LBNP

### Venous hemodynamics in µG: what do we currently know?

Venous hemodynamics have been found to be particularly sensitive to µG^31^. Fluid shifting to the cephalad region leads to the distension of cardiac chambers, increase in stroke volume, and accumulation of blood in the veins of the head and neck^2,32^. Increase in jugular vein, portal vein, and femoral vein diameter have been noted during spaceflight and µG analogs, which indicates venous pooling in cephalic, splanchnic, and pelvic regions^33^.

Beyond bulk fluid movement, the endothelial effects of venous stasis and pooling during spaceflight must be considered. It is known that the direction of flow and hence the direction of shear stress acting on the endothelium plays a crucial role for arterial wall remodeling processes and arterial thrombogenesis^34–37^; however, the role of the direction of venous of flow (reverse flow and stasis) and its potential impact on venous endothelial dysfunction is somewhat understudied. Considering the mechano-sensitivity of endothelial cells, it is possible that altered venous flow-mediated shear stress, as a result of altered venous hemodynamics in µG, may also play a role for inflammatory processes preceding venous wall remodeling and thrombogenesis^38^, as seen recently in an International Space Station (ISS) crewmember^17^.

The random discovery of an asymptomatic DVT in the jugular vein during an in-flight science experiment provides many causes for concern^16,17^. So far, there have been no known reports of this pathology in ground-based studies. It is unknown if DVT is not observed in ground-based studies because of lack of imaging/testing, or if there is a fundamental difference between fluid macro- and micro-circulation during spaceflight that increases the risk of blood clot formation.

### SANS: what do we currently know?

SANS is characterized by ocular changes and associated changes in the brain. The cardinal finding and current diagnostic criteria is uni- or bilateral optic disc edema of Frisen Grade 1 or more^39^. Additionally flattening of the globe, choroidal and retinal folds, cotton wool spots, and hyperoptic refractive error are often observed in those diagnosed with SANS^40^. Although SANS has only been identified in astronauts after spaceflight and has yet to be convincingly reproduced in a terrestrial analog, it bears some resemblance to terrestrial pathologies such as idiopathic intracranial hypertension (IIH) and normal pressure hydrocephalus (NPH)^39,41–44^. However, the exact etiology of SANS remains unknown, which makes terrestrial reproduction for the purposes of studying SANS countermeasures particularly difficult. Multiple hypotheses for the etiology of SANS exist and are explained in great detail in other work^15,39,45–47^. Here we present a summary of the hypothesized etiologies that may be mitigated by the application of LBNP.

The prevailing hypothesis on etiology of SANS is based on a slow but chronic cerebral and ocular overload of fluid and pressure associated with the systemic cephalad fluid shift in weightlessness. This in theory would constitute an increase in intracranial pressure (ICP). During bulk fluid movement, cerebrospinal fluid (CSF) may flow into orbital optic nerve space, and return flow could be impaired as a result of subarachnoid space remodeling, the body’s response to this overload of CSF^46^. Reduction of reabsorption of CSF and lymphatic drainage could cause cerebral edema leading to increased ICP^48^.

Alternatively, venous pooling/backflow could be a causative factor for SANS^46^. ICP seems to be governed by pressure in draining veins^49^. Internal jugular vein (IJV) diameter and blood flow decrease when moving from the supine to seated position under 1 G, while vertebral vein blood flow increases to maintain cerebral drainage^16,49^. This indicates that variation in posture (which lost in µG) has significant effects on cerebral drainage and therefore ICP. During spaceflight, the IJV is constantly distended due to the µG-induced headward fluid shift, which may have serious consequences for maintenance of cerebral drainage and ICP^16^. A recent in-flight study of 11 astronauts reported that 55% of the crew had stagnant and retrograde blood flow in their IJV^47^.

Some µG studies have documented that cerebral arterial diameters and blood flow velocity are auto-regulated and do not change significantly during spaceflight^39,50^, but, µG fluid shifts have been documented to cause jugular vein distension, also seen in head down tilt (HDT)^39^. The observed distension of the jugular vein and presumed increased jugular venous pressure during weightlessness could alter Starling forces and favor filtration of fluid into the interstitial space, further increasing ICP^15^.

The difficulty in determining the role of ICP in the complex pathology of SANS and cerebral perfusion and drainage is largely due to the invasive nature of this measurement making it unsuitable for spaceflight^39^. Despite enthusiasm from both the scientific and clinical communities, there are no reliable non-invasive ICP methods available at the time of writing. Invasive measurements have been performed during very short-term weightlessness in parabolic flight^51^, postural manipulation of the gravitational vector^52^, and simulated gravitational stress^21^. Based on this work, it has been hypothesized that ICP is not elevated to pathological levels in weightlessness, but rather stabilizes at levels between that of upright and supine terrestrial levels, thus never reaching normal upright levels to unload the brain^51,52^. Non-pathological but above normal upright levels of ICP without concomitant changes in intraocular pressure (IOP) causes a differential pressure increase across the lamina cribrosa that pushes on the eye, explaining the globe flattening observed in those diagnosed with SANS^15^.

### How could LBNP act as a simulation of gravitational stress to mitigate these pathologies?

When in an upright position under 1 G, gravity stresses the cardiovascular system by displacing blood and fluid toward the feet. To maintain blood pressure and perfusion of cranial structures, compensatory reflexes are elicited^3^. LBNP simulates the effect of gravitational stress in upright postures, but unlike true gravitational stress, it provokes an abrupt and uniform increase over the body, as opposed to the linear gradient of gravitational stress provided by upright posture in 1 G^53^. Despite these differences, LBNP may be a useful cardiovascular countermeasure; by reversing fluid shift and reducing venous return to the heart, compensatory arterial and cardiopulmonary baroreflexes are activated and maintained^8^.

Given the hypotheses for the etiology of SANS presented above, the use of LBNP to mitigate SANS relies on LBNP’s ability to combat cephalad fluid shifting, which has the potential to mitigate increases in ICP, IOP/ICP mismatch, and/or cerebral remodeling. One study found that ICP and internal jugular vein cross-sectional area (IJV CSA) increased during a transition from seated upright to 15° HDT, and LBNP was able to mitigate these increases: LBNP at −25 mmHg reduced ICP during HDT, whereas −50 mmHg reduced ICP and IJV CSA^21^. LBNP at −20 mmHg comfortably induced mechanical loading and desired fluid displacement, and was found to be ideal to lower ICP without impairing cerebral perfusion pressure^21^. Recently, LBNP used during bed rest was shown to reduce choroidal engouement (Lawley et al., 2018, ISGP Conference, Amsterdam, the Netherlands). In 12° HDT with −20 mmHg, LBNP was able to reduce increases in optic nerve sheath diameter, which is linked to ICP and intracranial CSF, which indicates the ability of LBNP to influence CSF^22^.

Taken together, LBNP can unload cerebral structures^21,22,28,51^, has a noticeable effect on cerebral circulation^7,49^ and on systemic cardiovascular adaptations and venous flow^2,7^. LBNP at −10 mmHg for 1 h decreased central venous pressure without affecting blood pressure or heart rate^54^, which indicates that it can activate the normal baroreceptors reflexes without causing undue stress on the body. Therefore, LBNP may maintain habitual endothelial shear stress^36^, counteracting endothelial dysfunction and venous pooling, and may also reduce plasma loss^18^, and thus mitigate orthostatic intolerance. LBNP was shown to increase blood flow in ten of 17 sessions during a recent ISS study, demonstrating its ability to alter hemodynamics during spaceflight^16^.

## How could LBNP be applied safely and effectively in µG?

### Feasibility of LBNP for use in space

LBNP has been in use by the American and Russian space agencies since the Shuttle and MIR eras, but the difficulty of application of LBNP, in terms of the safety risk of syncope in space when applying high pressures for short durations, and the inefficiency in regard to time usage when applying low pressures for long periods of time, coupled with an unknown dose response led to it falling out of use on the American and European sides^55^. However, the Russian cosmonauts continue to use a version of LBNP, the Chibis suit, to this day on the ISS. Other partners on the ISS use the Chibis infrastructure purely for research, as was done during the Fluid Shifts Experiment, but not as a countermeasure^12,18,56^.

To assuage safety concerns and to make LBNP more user friendly, lower pressures should be used for countermeasure purposes. As described above in Section “How could LBNP act as a simulation of gravitational stress to mitigate these pathologies?”, lower pressures (i.e. −10 mmHg to −20 mmHg) are seemingly most effective at mitigating the pathologies associated with loss of orthostatic tolerance and SANS. While higher pressures have been tested and occasionally found to be effective, the risk of syncope in space and loss of cerebral perfusion make high pressure applications both risky and impractical, as astronauts would need to be supervised under these higher pressures to monitor unwanted effects, which effectively negates the time benefit of high intensity/low duration application.

Recently, Dr. Petersen and team supported by NASA, have developed a mobile, wearable LBNP suit^28^. Further development of this effort will potentially facilitate LBNP as a feasible countermeasure for long duration spaceflight as it does not interfere with daily activities of the crewmembers^28^.

### What is the minimal effective dose of LBNP?

LBNP research in the early days of spaceflight went down to −100 mmHg^55^, which was likely to be both unnecessary and dangerous, and has likely contributed to the technique being discontinued throughout the ISS era.

Recent research shows that smaller magnitudes are effective, though the exact minimal effective dose for each identified fluid-shift-related pathology is unknown and likely to differ in what times and pressures are required^21,28^. Given the safety risk associated with bulk fluid redistribution in space, to convince flight surgeons and the crew that LBNP is effective and safe as a countermeasure, there should be a significant safety margin in how much pressure can be applied, and applied pressures and exposure times should be well below levels known to cause (pre-) syncope. High pressures are more likely to have the negative effects of syncope and reduced cerebral perfusion pressure, therefore instead of moving toward a high pressure, small time duration, research has trended in the long applications of low pressures.

## Research gaps and recommendations for future research

### SANS research gaps and recommendations

Over the course of this research, we identified series of knowledge gaps related to SANS that are listed below:

1. There is a lack of knowledge regarding the etiological mechanism and contributing risk factors for the ocular and cerebral changes associated with SANS. Testing sooner after landing, and using longer term follow-up studies to clarify reversibility may help reveal this etiology^39^.
2. There is a lack of appropriate diagnostic tools to measure and monitor changes in relevant parameters in a non-invasive manner. This is especially relevant for ICP, which requires an invasive port for measurements. Although there is currently some indication that in-flight invasive ICP will happen, and pre- and post-flight invasive ICP is already being done (humanresearchroadmap.nasa.gov), invasive investigations of ICP are unlikely to work in a long duration spaceflight scenario due to the high risk of adverse events, and the level of training and expertise needed to execute the gold standard procedures. Unfortunately, non-invasive ICP measurement tools are severely lacking^57^.
3. The reliability of ground-based analogs for simulating SANS etiology is unknown. There is some recent evidence that SANS can be simulated on Earth, as optic disc edema developed in 45% of people after 30 days of strict 6° HDT in 0.5% CO_2_ (ref. ^58^). However, this prevalence is higher than the prevalence of ~15% of astronauts, indicating a different underlying pathology or that HDT exaggerates fluid shift^47^.
4. There is a lack of countermeasures to prevent SANS or appropriate treatments to mitigate/reverse the changes observed in- and post-flight.

Therefore, the key recommendations for investigating SANS and effectively mitigating it are:

1. Understand the changes that result in the remodeling observed in SANS.
2. Better reproduction of SANS symptoms on Earth to test mitigation protocols.
3. The implementation of integrated mission protocols to search for multifactorial causes. The 1-year integrated mission protocol from NASA is likely to be helpful, as are ground-based integrated mission protocols.

### Venous stasis research gaps and recommendations

The NASA Human Research Roadmap (https://humanresearchroadmap.nasa.gov/) has identified gaps that are relevant to venous stasis and thrombosis, even though the recently identified DVT has not been formally incorporated into the research roadmap. We additionally identified gaps relevant to the recently identified DVT and possible contributing factors.

These knowledge gaps are as follows:

1. There is a lack of capability to mitigate select medical conditions during flight, which includes the recently identified venous thrombosis. Although it was treated with anti-coagulants^17^, this is a not a long-term solution that is appropriate for long duration spaceflight where Earth’s medical facilities are inaccessible. Moreover, pharmacokinetics and pharmaco-safety of anti-coagulant drugs used in µG are unknown and their use may impose severe health risks.
2. There is little information on the direction of systemic venous flow in µG available.
3. It is not known how µG affects venous endothelial shear stress.
4. There is no information on the potential impact of altered venous hemodynamics on venous endothelial inflammatory mechanisms available, though arterial endothelial dysfunction (flow mediated dilation) has been studied before in bed rest^59^.
5. It is not known if terrestrial venous thrombosis diagnostic tests such as the D-Dimer, pressure B-Mode Ultrasound, and Doppler Ultrasound, are reliable in µG.

Therefore, the recommendations for the study of venous stasis include:

1. Focus on non-invasive treatment strategies that can be used in space, such an anticoagulants and thrombolytics^48^.
2. In-depth analysis of venous hemodynamics in µG and the effects of LBNP.
3. Validation studies to validate DVT diagnostic tools in µG,

### LBNP research gaps and recommendations

Throughout this review, several gaps in the body of LBNP knowledge have been identified.

1. Due to the lack of widespread use of LBNP as an active countermeasure, the dose needed to treat is unknown and the safety protocols are yet to be validated.
2. It is currently unknown how LBNP affects venous endothelial shear stress, and without understanding the mechanism at hand it is impossible to know how LBNP may effectively mitigate the potentially negative effects of altered venous flow in µG.
3. It is unknown whether maintaining 1 G gravitational stress is favorable during long duration spaceflight, as opposed to letting adaptive changes occur, especially for partial gravity (ie. lunar, Martian).
4. There is a lack of understanding surrounding the mechanisms of bulk fluid movement during the use of LBNP.

Therefore, the recommendations for the study of LBNP as a countermeasure are as follows:

1. Continue to discern the dose needed to treat each pathology, i.e. SANS, orthostatic intolerance, alterations in venous hemodynamics. It is likely that different pathologies may need different protocols to be mitigated fully, due to the variety of mechanisms at work in each pathology. Although research such as the mobile LBNP suit is beginning to shed some light on these issues, they must be fully investigated before commencing in-flight use^28^.
2. More work needs to be done on LBNP in ground-based studies for SANS and venous thrombosis. Venous hemodynamics need to be studied in-depth in space and in spaceflight analogs to better characterize and quantify venous wall shear stress, endothelial dysfunction, early venous wall remodeling mechanisms, and circulating parameters to assess blood clotting, such as D-Dimer. To date, studies are finding favorable outcomes for the use of various countermeasures without fully understanding the etiology of why those countermeasures are beneficial. For example, LBNP and negative pressure respiration was found to be more favorable than just LBNP, but the exact mechanism for this remains largely unclear^60^.
3. Research into operational impacts in µG are necessary, especially for LBNP protocol development, as is predictive adaptation for various levels of gravity (i.e. lunar, Martian).
4. Microvascular responses show that LBNP redistributes fluids differently from standing under 1 G, therefore there is a critical need for well-developed modeling to understand the bulk fluid shift^53,61^.

## Conclusion

SANS and altered venous hemodynamics pose major threats to the health of astronauts during long duration spaceflight, and both pathologies are intricately linked to the lack of gravitational stress. LBNP may be a way to prevent negative outcomes during long duration spaceflight, by recreating or at least simulating to a reasonable level gravitational stress during spaceflight. However, to know how effective LBNP or other countermeasures are, significant research into the mechanism behind these pathologies, including an understanding of bulk fluid shift under µG, monitoring capabilities that are appropriate for spaceflight, and validation of ground-based analogs for modeling these pathologies are needed. Further research is needed into the cause of SANS and the extent of venous thrombosis risk before the full mitigative capacity of LBNP can be defined, and more robust work needs to be done on the implications of combining LBNP with other countermeasures.

## Data Availability

No datasets or codes were generated as a result of this research.

## References

[1] Arbeille P, Achaibou F, Fomina G, Pottier JM, Porcher M (1996). Regional blood flow in microgravity: adaptation and deconditioning. Med. Sci. Sports Exerc..

[2] Norsk, P. Adaptation of the cardiovascular system to weightlessness: surprises, paradoxes and implications for deep space missions. *Acta Physiol.*10.1111/apha.13434, 1–16 (2019). 10.1111/apha.13434 31872965

[3] Aubert AE, Beckers F, Verheyden B (2005). Cardiovascular function and basics of physiology in microgravity. Acta Cardiol..

[4] Petersen LG, Damgaard M, Petersen JCG, Norsk P (2011). Mechanisms of increase in cardiac output during acute weightlessness in humans. J. Appl. Physiol..

[5] Arbeille P (2017). Jugular and portal vein volume, middle cerebral vein velocity, and intracranial pressure in dry immersion. Aerosp. Med. Hum. Perform..

[6] Tomilovskaya E, Shigueva T, Sayenko D, Rukavishnikov I, Kozlovskaya I (2019). Dry immersion as a ground-based model of microgravity physiological effects. Front. Physiol..

[7] Levine B, Giller C, Lane L, Buckey J, Blomqvist C (1994). Cerebral versus systemic hemodynamics during graded orthostatic stress in humans. Circulation.

[8] Baisch F (2000). Cardiovascular response to lower body negative pressure stimulation before, during, and after space flight. Eur. J. Clin. Invest..

[9] Convertino VA (2009). Status of cardiovascular issues related to space flight: implications for future research directions. Respir. Physiol. Neurobiol..

[10] Custaud MA (2000). No effect of venoconstrictive thigh cuffs on orthostatic hypotension induced by head-down bed rest. Acta Physiol. Scand..

[11] Hargens AR, Richardson S (2009). Cardiovascular adaptations, fluid shifts, and countermeasures related to space flight. Respir. Physiol. Neurobiol..

[12] Yarmanova EN, Kozlovskaya IB, Khimoroda NN, Fomina EV (2015). Evolution of Russian microgravity countermeasures. Aersp. Med. Hum. Perform..

[13] Sun X-Q (2002). Effect of lower body negative pressure against orthostatic intolerance induced by 21 days head-down tilt bed rest. Aviat. Space Environ. Med..

[14] Watenpaugh, D. E. & Hargens, A. R. in *Handbook of Physiology, Environmental Physiology* (eds Fregly, M. & Blatteis, C.) 631–674 (Oxford University Press, 1996).

[15] Stenger, M. B. et al. Evidence report: risk of spaceflight associated neuro-ocular syndrome (SANS). *NTRS - NASA Technical Reports Server*. https://ntrs.nasa.gov/citations/20180000936 (2017).

[16] Marshall-Goebel K (2019). Assessment of jugular venous blood flow stasis and thrombosis during spaceflight. JAMA Netw. Open.

[17] Auñón-Chancellor SM, Pattarini JM, Moll S, Sargsyan A (2020). Venous thrombosis during spaceflight. N. Engl. J. Med..

[18] Crystal GJ, Salem MR (2015). Lower body negative pressure: historical perspective, research findings, and clinical applications. J. Anesth. Hist..

[19] Watenpaugh DE (2007). Lower body negative pressure exercise plus brief postexercise lower body negative pressure improve post-bed rest orthostatic tolerance. J. Appl. Physiol..

[20] Güell A, Braak L, le Traon AP, Gharib C (1990). Cardiovascular deconditioning during weightlessness simulation and the use of Lower Body Negative Pressure as a countermeasure to orthostatic intolerance. Acta Astronaut..

[21] Petersen LG (2019). Lower body negative pressure to safely reduce intracranial pressure. J. Physiol..

[22] Marshall-Goebel K (2017). Lower body negative pressure reduces optic nerve sheath diameter during head-down tilt. J. Appl. Physiol..

[23] Watkins W, Hargens AR, Seidl S, Clary EM, Macias BR (2017). Lower-body negative pressure decreases noninvasively measured intracranial pressure and internal jugular vein cross-sectional area during head-down tilt. J. Appl. Physiol..

[24] Halliwill JR, Lawler LA, Eickhoff TJ, Joyner MJ, Mulvagh SL (2017). Reflex responses to regional venous pooling during lower body negative pressure in humans. J. Appl. Physiol..

[25] Arbeille P (2012). Aortic, cerebral and lower limb arterial and venous response to orthostatic stress after a 60-day bedrest. Eur. J. Appl. Physiol..

[26] Lee SMC (2014). WISE-2005: countermeasures to prevent muscle deconditioning during bed rest in women. J. Appl. Physiol..

[27] Smith SM (2008). WISE-2005: supine treadmill exercise within lower body negative pressure and flywheel resistive exercise as a countermeasure to bed rest-induced bone loss in women during 60-day simulated microgravity. Bone.

[28] Petersen LG (2019). Mobile lower body negative pressure suit as an integrative countermeasure for spaceflight. Aerosp. Med. Hum. Perform..

[29] Fiebig, L. et al. Effectiveness of resistive exercise countermeasures in bed rest to maintain muscle strength and power – a systematic review. *Front. Physiol.* **9** (2018).

[30] Petersen N (2016). Exercise in space: The European Space Agency approach to in-flight exercise countermeasures for long-duration missions on ISS. Extrem. Physiol. Med..

[31] Kotovskaia AR, Fomina GA (2010). The features of adaptation and disadaptation of the human cardiovascular system in the space flight conditions. Fiziol. Cheloveka.

[32] Norsk P, Asmar A, Damgaard M, Christensen NJ (2015). Fluid shifts, vasodilatation and ambulatory blood pressure reduction during long duration spaceflight. J. Physiol..

[33] Arbeille P, Provost R, Zuj K, Vincent N (2015). Measurements of jugular, portal, femoral, and calf vein cross-sectional area for the assessment of venous blood distribution with long duration spaceflight (Vessel Imaging Experiment). Eur. J. Appl. Physiol..

[34] Gimbrone MA, García-Cardeña G (2013). Vascular endothelium, hemodynamics, and the pathobiology of atherosclerosis. Cardiovasc. Pathol..

[35] Bryan MT (2014). Mechanoresponsive networks controlling vascular inflammation. Arterioscler. Thromb. Vasc. Biol..

[36] Johnson BD, Mather KJ, Wallace JP (2011). Mechanotransduction of shear in the endothelium: basic studies and clinical implications. Vasc. Med..

[37] Chistiakov DA, Orekhov AN, Bobryshev YV (2017). Effects of shear stress on endothelial cells: go with the flow. Acta Physiol..

[38] Chiu J-J, Chien S (2011). Effects of disturbed flow on vascular endothelium: pathophysiological basis and clinical perspectives. Physiol. Rev..

[39] Lee AG, Mader TH, Gibson CR, Brunstetter TJ, Tarver WJ (2018). Space flight-associated neuro-ocular syndrome (SANS). Eye.

[40] Mader TH (2011). Optic disc edema, globe flattening, choroidal folds, and hyperopic shifts observed in astronauts after long-duration space flight. Ophthalmology.

[41] Qvarlander S, Sundström N, Malm J, Eklund A (2013). Postural effects on intracranial pressure: modeling and clinical evaluation. J. Appl. Physiol..

[42] Roberts DR (2017). Effects of spaceflight on astronaut brain structure as indicated on MRI. N. Engl. J. Med..

[43] Petersen LG, Ogoh S (2019). Gravity, intracranial pressure, and cerebral autoregulation. Physiol. Rep..

[44] Roberts DR, Petersen LG (2019). Studies of hydrocephalus associated with long-term spaceflight may provide new insights into cerebrospinal fluid flow dynamics here on earth. JAMA Neurol..

[45] Wojcik P, Kini A, Al Othman B, Galdamez LA, Lee AG (2020). Spaceflight associated neuro-ocular syndrome. Curr. Opin. Neurol..

[46] Mader TH (2020). Letter: brain physiological response and adaptation during spaceflight. Neurosurgery.

[47] Marshall-Goebel K, Damani R, Bershad EM (2019). Brain physiological response and adaptation during spaceflight. Neurosurgery.

[48] Agrawal A, Pacheco-Hernandez A, Moscote-Salazar LR (2019). Letter: Neurosurgery and Manned Spaceflight. Clin. Neurosurg..

[49] Ogoh S (2016). Coupling between arterial and venous cerebral blood flow during postural change. Am. J. Physiol. Regul. Integr. Comp. Physiol..

[50] Iwasaki K (2007). Human cerebral autoregulation before, during and after spaceflight. J. Physiol..

[51] Lawley JS (2017). Effect of gravity and microgravity on intracranial pressure. J. Physiol..

[52] Petersen LG, Petersen JCG, Andresen M, Secher NH, Juhler M (2016). Postural influence on intracranial and cerebral perfusion pressure in ambulatory neurosurgical patients. Am. J. Physiol. Regul. Integr. Comp. Physiol..

[53] Breit GA, Watenpaugh DE, Ballard RE, Murthy G, Hargens AR (1993). Regional cutaneous microvascular flow responses during gravitational and LBNP stresses. Physiologist.

[54] Hirsch, A. T., Levenson, D. J., Cutler, S. S., Dzau, V. J. & Creager, M. A. Regional vascular responses to prolonged lower body negative pressure in normal subjects. *Am. J. Physiol*. **257**, H219–H225 (1989). 10.1152/ajpheart.1989.257.1.H219 2750938

[55] Campbell MR, Charles JB (2015). Historical review of lower body negative pressure research in space medicine. Aerosp. Med. Hum. Perform..

[56] Stenger, M. et al. Fluid shifts. *NTRS - NASA Technical Reports Server*. https://ntrs.nasa.gov/citations/20150021479 (2016).

[57] Raboel PH, Bartek J, Andresen M, Bellander BM, Romner B (2012). Intracranial pressure monitoring: Invasive versus non-invasive methods-A review. Crit. Care Res. Pract..

[58] Laurie SS (2019). Optic disc edema after 30 days of strict head-down tilt bed rest. Ophthalmology.

[59] dela Paz NG, D’Amore PA (2009). Arterial versus venous endothelial cells. Cell Tissue Res..

[60] Baranov VM (2003). Comparative evaluation of several methods preventing orthostatic disorders during simulation of the end-of-space-mission factors. Aviakosm. Ekol. Med..

[61] Watenpaugh DE (2004). Human cutaneous vascular responses to whole-body tilting, G z centrifugation, and LBNP. J. Appl. Physiol..

[62] Anderson AP, Butterfield JS, Subramanian PS, Clark TK (2018). Intraocular pressure and cardiovascular alterations investigated in artificial gravity as a countermeasure to spaceflight associated neuro-ocular syndrome. J. Appl. Physiol..

[63] Chen JJ (2015). Causes and prognosis of visual acuity loss at the time of initial presentation in idiopathic intracranial hypertension. Invest. Opthalmol. Vis. Sci..

[64] Kuriyama N (2008). Retrograde jugular flow associated with idiopathic normal pressure hydrocephalus. Ann. Neurol..

